# Healthcare providers’ perception of advance care planning for patients with critical illnesses in acute-care hospitals: a cross-sectional study

**DOI:** 10.1186/s12904-021-00900-5

**Published:** 2022-01-07

**Authors:** Kanako Yamamoto, Yuki Yonekura, Kazuhiro Nakayama

**Affiliations:** 1 Department of Critical Care Nursing, Graduate School of Nursing Science, St. Luke’s International University, 10-1, Akashi-cho, Tokyo, Japan; 2 Department of Nursing Informatics, Graduate School of Nursing Science, St. Luke’s International University, 10-1, Akashi-cho, Tokyo, Japan

**Keywords:** Acute-care hospital, Advance care planning, Critical care, Intensive care unit, Advance directives

## Abstract

**Background:**

In acute-care hospitals, patients treated in an ICU for surgical reasons or sudden deterioration are treated in an outpatient ward, ICU, and other multiple departments. It is unclear how healthcare providers are initiating advance care planning (ACP) for such patients and assisting them with it. The purpose of this study is to clarify healthcare providers’ perceptions of the ACP support provided to patients receiving critical care in acute-care hospitals.

**Methods:**

A cross-sectional study was conducted using questionnaires. In this study, 400 acute-care hospitals with ICUs in Japan were randomly selected, and 1490 subjects, including intensivists, surgeons, ICU nurses, surgical floor nurses, and surgical outpatient nurses, participated. Survey items examined whether ICU patients received ACP support, the participants’ degree of confidence in providing ACP support, the patients’ treatment preferences, and the decision-making process, and whether any discussion was conducted on change of values.

**Results:**

Responses were obtained from 598 participants from 157 hospitals, 41.4% of which reportedly supported ACP provision to ICU patients. The subjects with the highest level of ACP understanding were surgeons (45.8%), and differences in understanding were observed across specialties (*P* < 0.001). Among the respondents, physicians and nurses expressed high levels of confidence in providing ACP support to patients requiring critical care. However, 15.2% of all the subjects mentioned that they would not attempt to resuscitate the patients. In addition, 25.7% of the participants handed over patients’ values to other departments or hospitals, whereas 25.3% handed over the decision-making process.

**Conclusions:**

Among the participating hospitals, 40% provided ACP support to patients receiving critical care. The low number is possibly because support providers lack understanding of the content of patients’ ACP or about how to support and use ACP. Second, it is sometimes too late to start providing ACP support after ICU admission. Third, healthcare providers differ in their perception of ACP, widely considered an ambiguous concept. Finally, in acute-care hospitals with different healthcare settings, it is necessary to confirm and integrate the changes in feelings and thoughts of patients.

**Supplementary Information:**

The online version contains supplementary material available at 10.1186/s12904-021-00900-5.

## Background

Patients receiving critical care often experience the sudden onset of life-threatening symptoms. Advance care planning (ACP) empowers patients in the final stage of their lives to spend their days according to their wishes. Advance care planning can include advance directives (ADs) as for example do not attempt resuscitation (DNAR) and living wills. ACP is a process that concretises ADs by discussing patients’ wishes regarding treatment, values, goals, and preferences [1]. There are differences in the way various countries approach the ACP and how they respond to the wishes of patients. In Europe and the United States, the wishes of patients are protected by law through the documentation of ADs. In Japan, however, the ACP is only a recommendation in the guidelines and is not legally enforceable [2].

Previous ACP studies have been conducted in patients with advanced cancer and in elderly patients living in nursing homes [3, 4]. However, currently, only inadequate ACP support is provided to patients treated in intensive care units (ICUs) [5, 6]. This may be the result of the unclear boundary between life-saving and life-prolonging strategies in the critical care process. In addition, it is challenging to discuss ACP among ICU patients, because the ICU is widely considered a place to receive life-saving treatment [7]. In general, the goal of ACP is quality end-of-life care. It is considered that predicting the patient’s prognosis and understanding that the patient is in the terminal stage will lead to the initiation or promotion of ACP support for the patient. However, one of the problems faced by intensive care physicians is in an accurate prediction of such patients’ prognoses [8]. It is reported that 50–60% of patients with DNAR in the ICU remained alive till their discharge [9, 10]. This suggests that aggressive treatment may not be futile, even in patients whose recovery may be deemed difficult. In addition, healthcare providers remain indifferent to ACP because the ICU may be widely recognized as a place to provide life-saving treatment.

Moreover, since the ICU is where patients undergoing sudden changes in their disease conditions are treated, it is difficult in many cases to confirm the patients’ wishes regarding treatment, and the implementation of ACP in the ICU is complex too. ICU patients are often incapable of making decisions themselves, and a surrogate decision-maker is required to provide the necessary explanation and consent for DNAR [11–13]. Therefore, healthcare providers and surrogate decision-makers often have compelling reasons to decide on transition from lifesaving to end-of-life medical care without any information on the patient’s wishes; the DNAR order speeds up this transition [14–16]. Another reason for the difficulty in supporting ACP for patients treated in acute-care hospitals and ICUs is the involvement of multiple practitioners. Patients who receive temporary surgical treatment or emergency transport at an acute-care hospital likely have more than one primary care physician, as well as multiple attending physicians, at the hospital. Moreover, patients with multiple diseases receive treatment from more than one expert physician. A study on patients with poor outcomes admitted to the ICU revealed conflicts among intensivists, ICU nurses, cardiovascular surgeons, cardiothoracic surgeons, and neurosurgeons in determining the patients’ treatment goals [17]. In this study, 60% of intensivists and surgeons had conflicting opinions about determining patients’ end-of-life care preferences. One reason for the conflict is the unique ICU management system, with more than one physician making decisions about a patient’s treatment. In some circumstances, the provision of ACP support to ICU patients, which usually involves only the patient and their primary physician, differs significantly from the recommended process. An essential aspect of ACP is the process in which surrogate decision-makers and healthcare providers discuss and share DNAR and ADs regarding how patients live, what they value, and what they desire [18]. However, among patients receiving critical care, the discussion in which healthcare providers and patients share treatment values is difficult, and DNAR decisions are often the main focus [13–16]. Further, in acute-care hospitals, critically ill patients are treated by multiple healthcare providers, and treatments of a patient are generally not confined to the outpatient, general ward, and ICU settings. In such hospitals, the manner in which a healthcare provider confirms and takes over a patient’s wishes is considered an important aspect of the patient’s treatment process. However, to date, no studies have clarified the status of ACP support provided to patients receiving critical care from the perspectives of multiple occupations and departments. By clarifying these findings, one may realise important ways to support the provision of patient-centred care to such patients in acute-care hospitals. In addition, it may be possible to clarify how to support patients based on their treatment processes and the continuity of their care. Thus, the purpose of this study is to clarify healthcare providers’ perception of ACP support for patients receiving critical care in acute-care hospitals.

## Methods

### Study design

A cross-sectional study was conducted in acute-care hospitals with ICUs in Japan. The current study is a sub-study on decision-making support in acute-care hospitals with ICUs in Japan, focusing on how ACP support and discussion start for patients receiving critical care in acute-care hospitals. As part of the study, a survey was carried out by distributing an anonymous self-reported questionnaire during August to December 2019.

### Participants

The criteria for participant selection were as follows: (1) physicians working in an ICU as an intensive care specialist, (2) surgeons providing consultations to the ICU during the perioperative period (cardiovascular surgeons, thoracic surgeons, gastrointestinal surgeons, neurosurgeons, etc.), (3) nurses with at least 3 years of ICU experience, (4) nurses with at least 3 years of experience working in surgical wards, and (5) nurses with at least 3 years of experience working in surgical outpatient clinics. Participants meeting any of the following criteria were excluded: (1) resident physicians and (2) nurses in their first or second year of employment.

In this study, statistical data were analysed using analysis of variance. An estimated correlation ratio of 0.05 and a detection power of 0.8 were set, and a total of 350 subjects, with 70 subjects from each department (ICU, Surgical, Outpatient), were considered as the target sample. The research targeted 400 randomly selected hospitals with ICUs in Japan under a 30% response rate assumption. Therefore, approximately 1–3 physicians, 5 ICU nurses, 5 surgical floor nurses and 1–3 surgical outpatient nurses were sampled per facility. We asked the director of a hospital to cooperate in this study The number of samples were a total of 1520 comprising 240 full-time intensivists, 240 surgeons, 400 ICU nurses, 400 floor nurses, and 240 outpatient nurses. Of these, the following were granted informal consent for surveys: 155 intensivists, 127 surgeons, 555 ICU nurses, 422 floor nurses, and 231 outpatient nurses and a total of 170 hospitals. The total number was 1490.

### Data collection and statistical analysis

Data collection was carried out by mailing a survey request form and a sample questionnaire to the managers of each department of the target hospitals. The facilities recruited were added in the order in which the survey approval was obtained, and the questionnaire was sent to the participants. Next, the questionnaire with a return envelope were sent to the participants. The survey’s purpose and method and the treatment of personal information were explained in writing to each participant. After completing the questionnaire, the participants sealed the envelope and sent it back by post.

### Survey questionnaire development

A questionnaire was developed for this study, with reference to guidelines on end-of-life medical treatment of emergency/critical care in Japan [18] and to the educational material for healthcare providers on ACP [19]. The questionnaires included questions testing the participants’ knowledge of ACP and the current status of support for ACP in their respective hospitals. The appropriateness of the items was discussed and assessed by the authors of this study. Regarding the easy-to-answer questionnaire, about 10 healthcare providers were asked to answer it (Additional file 1). The main outcomes of this survey clarified whether or not ACP support was provided to prospective ICU patients. Besides, questions of the survey examined how to obtain consent for DNAR, how often to obtain confirmation, how and when to confirm the wishes of prospective ICU patients, how to collaborate with other departments and institutions, and knowledge of ACP. Participants’ basic attributes, such as their age, gender, years of work experience, and designation, were also collected.

Physicians and nurses were asked to describe their professional qualifications, while floor nurses and outpatient nurses noted the department to which they belonged, and ICU nurses, intensivists, and surgeons were asked to categorise the ICU management system they operated within. The ICU management systems were categorized into the following three groups for the purpose of this study: (1) A closed-ICU is a facility where only intensivists make decisions on all the patients’ treatment strategies. (2) In a semi-ICU, intensivists intervene for all patients in the ICU or for patients in need (they work with the patient’s primary physician). (3) An ICU without an intensivist was called an open-ICU (open format ICU).

### Data analysis

IBM SPSS Statistics Ver. 25.0 software was used for statistical analysis. After basic statistical analysis, normality was confirmed and descriptive statistics were performed. The difference in perceptions by occupation and the average difference between departments were obtained. Further, the existence of any significant difference among five participants having different occupations and belonging to various departments was examined using one-way analysis of variance.

Tukey’s multiple comparison test was performed whenever significant differences less than 5% were found. Further, when equal group variance could not be assumed, Welch’s test was performed, and when a significant difference at < 5% was observed, Game-Howell’s multiple comparisons were performed. The t-test was conducted to analyse two groups with different occupations and departments. Finally, the χ2 test was performed for the nominal scale, and the significance level was set as < 5% (two-sided test). All statistical analyses were performed by supervising statisticians.

### Ethical considerations

Participants were provided written explanations of the survey’s purpose, research methods used, details of participating in and withdrawing from the survey, protection of personal information, and data management methods used in the study. The collected data were managed separately, and ID numbers and a correspondence table were created to ensure that individual participants could not be identified from the data. Further, data such as basic attributes were coded to maintain participant anonymity. Since participation was anonymous and voluntary, the completed questionnaires sent in by participants themselves were considered as consent.

## Results

### Participant overview

Completed questionnaires were returned by 124 intensivists (response rate, 80.0%), 59 surgeons (46.5%), 183 ICU nurses (33.0%), 123 floor nurses (29.1%), and 109 outpatient nurses (47.2%) in 157 institutions (92.4%). Table 1 summarises the demographic details of participants. Most of the participants belonged to the semi-closed ICU management system (210 participants or 57.4%). From the surgery departments, 23 (39.0% of the total) cardiovascular surgeons, 22 (37.3%) gastrointestinal surgeons, and 10 (16.9%) neurosurgeons participated in the study.

**Table 1:** Demographic details of survey respondents

Characteristic	Intensivist		Surgeon		ICU nurse		Floor nurse		Outpatient nurse	
	n	%	n	%	n	%	n	%	n	%
Gender										
Male	106	85.5	57	96.6	51	27.9	14	11.4	2	1.8
Female	18	14.5	2	3.4	132	72.1	109	88.6	107	98.2
Position										
Manager	38	30.6	17	28.8	14	7.7	17	13.8	12	11
Chief	28	22.6	20	33.9	29	15.8	19	15.4	13	11.9
Staff	58	46.8	22	37.3	140	76.5	87	70.7	85	77.1
Clinical department										
Gastroenterology			22	37.3			67	54.5	38	34.9
Cardiovascular			23	39.0			31	25.2	38	34.9
Thoracic surgery			3	5.1						
Neurosurgery			10	16.9			16	13.0	21	19.2
Other			1	1.7			9	5.1	12	11.0
Specialist qualifications										
Certified nurse					19	10.4	17	13.8	13	11.9
Certified nurse specialist					5	2.7	6	4.9	2	1.8
Nurse practitioner					1	0.5	0	0.0	0	0.00
None					158	86.3	100	81.3	94	86.2
Type of ICU										
Closed-ICU	28	22.6	3	5.1	21	11.5				
Semi-Closed ICU	79	63.7	42	71.2	89	48.6				
Open-ICU	17	13.7	14	23.7	73	39.9				
Age, mean ± SD	45.7 ± 10.0		46.3 ± 9.4		35.3 ± 7.3		35.6 ± 8.6		42.9 ± 7.8	
Years of work experience, mean ± SD	20.3 ± 9.2		21.0 ± 9.5		12.6 ± 6.9		12.6 ± 7.4		19.0 ± 8.0	

*ICU* Intensive care unit; *SD* Standard deviation

NOTES. Total numbers of intensivists, *n* = 124; surgeons, *n* = 59; ICU nurses, *n* = 183; floor nurses, *n* = 123; outpatient nurses, *n* = 109.

A closed ICU is a facility where only intensivists make decisions on all the treatment strategies for patients.

In semi-closed ICUs, intensivists intervene for all patients in the ICU or the patients in need.

An ICU without an intensivist is referred to as an open ICU

### Healthcare professionals’ perception of ACP support

As depicted in Tables 2, 25 intensivists (20.2%), 9 surgeons (15.3%), 22 ICU nurses (12.0%), 14 floor nurses (11.4%), and 17 outpatient nurses (15.6%) reported having provided ACP support to patients in, or before entering, the ICU. In addition, 65 of the 157 participating hospitals offered ACP support to patients. Although there was no common characteristic among the prominent participants in each job category, intensivists and surgeons were more prevalent than others. When asked what they said when ICU patients moved to general ward or outpatient, the most frequent answer was “medical information and discharge plans”; this was transitioned by 110 (91.3%) intensivists, 50 (84.7%) surgeons, and 144 (78.7%) ICU nurses, while 32 (27.2%) intensivists, 8 (13.6%) surgeons, and 50 (27.3%) ICU nurses transitioned their patients’ life worth and values (*P* = 0.006).

**Table 2:** Status of the introduction of ACPs for patients in ICUs

	Intensivist		Surgeon		ICU nurse		Floor nurse		Outpatient nurse		*P* value
	n	%	n	%	n	%	n	%	n	%	
ACP support											
I did.	25	20.2	9	15.3	22	12	14	11.4	17	15.6	0.0189
Who supported the ACP?											
Intensivists	42	73.7	1	5.9	15	8.2	0	0.0	0	0.0	
Surgeons	22	38.6	11	64.7	21	11.5	13	10.6	9	8.3	
Nurses	7	12.3	2	11.8	30	16.4	6	4.9	3	2.8	
Palliative care team	3	5.3	1	5.9	6	3.3	4	3.3	2	1.8	
Transition of patients’ treatment preferences and decisions ^a^											
Medical information and discharge plans	110	88.7	50	84.7	144	78.7	86	69.9	70	64.2	< 0.001
Treatment desired by the patient	77	62.1	30	50.8	128	69.9	84	68.3	64	58.7	0.035
Desired place for recovery	32	25.8	20	33.9	49	26.8	67	54.5	51	46.8	< 0.001
Patient’s values, and something to live for	32	25.8	8	13.6	50	27.3	31	25.2	33	30.3	0.218
Process leading to treatment decision	37	29.8	8	13.6	54	29.5	27	22.0	26	23.9	0.081
Surrogate decision-maker	48	38.7	10	16.9	65	35.5	38	30.9	19	17.4	< 0.001
I won’t give you any information	4	3.2	0	0.0	5	2.7	3	2.4	6	5.5	0.377
Other	4	3.2	0	0.0	8	4.4	1	0.8	1	0.9	

*ICU* Intensive care unit; *ACPs* Advance care plans

NOTES. Total numbers of intensivists, *n* = 124; surgeons, *n* = 59; ICU nurses, *n* = 183; floor nurses, *n* = 123; outpatient nurses, *n* = 109.

^a^ Include duplicate answer

The degree of understanding of the use of ACPs was strongest among intensivists (21% “often understanding”, 4% “always understanding”) (*P* = 0.097) (Table 3). Further, 27 surgeons (45.8%) were able to select the correct items for ACP knowledge, with a significant difference (*P* < 0.001). The degrees of confidence of various participants in supporting ACP provision to patients and their families were 48.8 ± 26.5, 46.9 ± 27.7, 34.6 ± 22.7, 38.5 ± 21.3, and 36.8 ± 27.1 for intensivists, surgeons, ICU nurses, floor nurses, and outpatient nurses, respectively (*P* = 0.016).

**Table 3:** Medical professionals’ understanding and awareness of ACPs

	Intensivist		Surgeon		ICU nurse		Floor nurse		Outpatient nurse		*P* value
	n	%	n	%	n	%	n	%	n	%	
Do you understand ACPs?											
Never	6	4.8	6	10.2	30	16.4	22	17.9	20	18.3	0.097
Occasionally	31	25.0	25	43.1	75	41.0	40	32.5	35	32.1	
Sometimes	56	45.2	21	36.2	53	29.0	46	37.4	36	33.0	
Often	26	21.0	5	8.6	17	9.3	10	8.1	15	13.8	
Always	5	4.0	1	1.7	4	2.2	0	0.0	1	0.9	
No response	0	0.0	0	0.0	4	2.2	5	4.1	2	1.8	
Which of the following statements do you recognise as the correct content of ACPs? ^a^											
It is carried out for people whose prognosis is that they will die within one year.	14	11.3	11	19.0	16	8.7	18	14.6	8	7.3	< 0.001
The healthcare provider selects the most appropriate person as the proxy decision-maker.	12	12.5	7	11.9	13	7.1	12	9.8	10	9.2	0.169
Healthcare providers ask patients about their treatment preferences; they also ask the patients to decide.	75	61.0	40	69.0	91	49.7	53	43.1	36	33.0	< 0.001
Healthcare providers must always inform patients of the expected course of illness and life expectancy (prognosis).	45	36.3	16	27.6	63	34.4	32	26.0	46	42.2	< 0.001
Decisions once considered should be adhered to, and treatments’ intentions should remain unchanged.	1	0.8	1	1.7	2	1.1	3	2.4	2	1.8	0.227
Correct answer	42	33.9	27	45.8	62	33.9	35	28.5	20	18.3	< 0.001
Degree of confidence in providing ACPs support to patients/families (range 0–100), mean ± SD	48.8 ± 26.5		46.9 ± 27.7		34.6 ± 22.7		38.5 ± 21.3		36.8 ± 27.1		0.016

*ICU* Intensive care unit; *ACPs* Advance care plans; *SD* Standard deviation

NOTES*.* Total numbers of intensivists, *n* = 124; surgeons, *n* = 59; ICU nurses, *n* = 183; floor nurses, *n* = 123; outpatient nurses, *n* = 109.

The table depicts one-way analysis of variance results.

^a^ Include duplicate answer

Further, several intensivists (41, 33.1%), surgeons (23, 39.0%), ICU nurses (58, 31.7%), floor nurses (87, 70.7%), and outpatient nurses (76, 69.7%) responded that the patient selected the surrogate decision-maker (*P* = 0.127) (Table 4). The most preferred surrogate decision-maker was someone from among the patient’s family (*P* < 0.001). In addition, 20 intensivists (16.1%), 8 surgeons (13.6%), 21 ICU nurses (11.5%), 32 floor nurses (26.0%), and 10 outpatient nurses (9.2%) said that patients could perform DNAR confirmation (often and always) (*p* < .001). According to intensivists, surgeons, and ICU nurses, DNAR confirmation was commonly performed ‘at the time of disease worsening’. In addition, 32 intensivists (25.9%), 11 surgeons (18.7%), 53 ICU nurses (29.0%), 12 floor nurses (9.8%), and 8 outpatient nurses (7.3%) agreed that re-discussed DNAR should be performed frequently, or absolutely, whenever the patient’s condition changed (*P* < .001).

**Table 4:** Results of discussing DNAR with critical care patients

	Intensivist		Surgeon		ICU nurse		Floor nurse		Outpatient nurse		*P* value
	n	%	n	%	n	%	n	%	n	%	
Do you check DNAR with a patient?											0.080
Never	33	26.6	27	45.8	84	45.9	30	24.4	46	42.2	
Occasionally	47	37.9	16	27.1	52	28.42	30	24.4	35	32.1	
Sometimes	24	19.4	8	13.6	25	13.66	31	25.2	16	14.7	
Often	15	12.1	6	10.2	16	8.743	22	17.9	10	9.2	
Always	5	4.0	2	3.4	5	2.732	10	8.1	0	0.0	
Non response	4	3.2	0	0.0	1	0.546	0	0.0	4	3.7	
Timing ^a^											
Admitted in the ICU	40	32.3	7	11.9	49	26.8	0	0.0	0	0.0	
When condition worsens	69	55.6	25	42.9	89	48.6	96	78.0	65	59.6	
Do not check with the patient	17	13.7	15	25.4	54	29.5	17	13.8	3	2.8	
At the time of hospital admission	39	31.5	6	10.2	40	21.9	33	26.8	18	16.5	
During the first outpatient visit	0	0.0	0	0.0	0	0.0	19	15.4	6	5.5	
Other	9	7.3	3	5.1	14	7.7	4	3.3	13	11.9	
Do you recheck DNAR when the patient’scondition improves?											0.086
Never	20	16.1	12	22.2	21	11.5	51	41.5	52	47.7	
Occasionally	49	39.5	19	35.2	53	29.0	34	27.6	24	22.0	
Sometimes	21	16.9	12	22.2	47	25.7	24	19.5	14	12.8	
Frequently	24	19.4	4	7.4	40	21.9	8	6.5	7	6.4	
Absolutely	8	6.5	7	13.0	13	7.1	4	3.3	1	0.9	
No response	2	1.6	5	8.5	9	4.9	2	1.6	10	9.2	
Who decides on selecting surrogate decision-maker? ^a^											
Intensivists	22	17.7	7	11.9	9	4.9	1	0.8	0	0.0	< 0.001
Surgeons	67	54.0	28	47.5	35	19.1	17	13.8	34	31.2	< 0.001
ICU nurses	33	26.6	16	27.1	32	17.5	3	2.4	0	0.0	< 0.001
Patients	41	33.1	23	39.0	58	31.7	87	70.7	76	69.7	0.127
Patients’ families	91	73.4	43	72.9	166	90.7	103	83.7	72	66.1	< 0.001
Floor nurses	0	0.0	0	0.0	0	0.0	0	0.0	14	12.8	
Outpatient nurses	0	0.0	0	0.0	0	0.0	0	0	18	16.5	
Other	2	1.6	1	1.7	4	2.2	6	5	1	0.9	

*DNAR* Do not attempt resuscitation; *ICU* Intensive care unit

*NOTES.* Total numbers of intensivists, *n* = 124; surgeons, *n* = 59; ICU nurses, *n* = 183; floor nurses, *n* = 123; outpatient nurses, *n* = 109.

The table depicts one-way analysis of variance results.

^a^ Include duplicate answer

### Provision of decision-making support to patients and performance of cross-departmental information-sharing by healthcare providers

To the question whether patients admitted to the ICU might be asked about their wishes to receive treatment, 32 (25.8%) intensivists, 11 (18.6%) surgeons, and 64 (35.0%) ICU nurses answered that the patients were asked often or always (*p* = 0.148) (Table 5). In addition, the confirmation of treatment intention was most commonly performed when a new treatment was considered for all participants, followed by the time of disease progression. Among all healthcare providers, surgeons were the ones who most frequently confirmed patients’ intentions. In the treatment intention content confirmed to the patient, the selection of treatment was the most frequent for all participants – 80–90%. This was followed by the progress in disease prediction. The patients’ values were ascertained by 72 (58.1%) intensivists, 27 (45.8%) surgeons, and 71 (38.8%) ICU nurses (*P* = 0.006). Fully 53 (42.7%) intensivists, 35 (60.4%) surgeons, and 79 (44.6%) ICU nurses responded that their treatment always/often reflected the patients’ wishes (*p* = 0.378).

**Table 5:** Confirmation of the treatment preferences of patients in ICUs

	Intensivist		Surgeon		ICU nurse		*P* value
	n	%	n	%	n	%	
Do you confirm the patient’s intention regarding treatment?							0.143
Never	13	10.5	4	6.8	12	6.6	
Occasionally	44	35.5	25	42.4	58	31.7	
Sometimes	34	27.4	19	32.2	48	26.2	
Often	20	16.1	10	16.9	43	23.5	
Always	12	9.7	1	1.7	21	11.5	
When do you confirm the patient’s intention regarding treatment? ^a^							
On admission to the ICU	57	46.0	21	18.3	69	37.7	
When condition worsens	32	25.8	9	7.5	59	32.2	
At the time of disease improvement	79	63.7	37	31.2	111	60.7	
Before starting a new treatment	80	64.5	44	38.7	126	68.9	
Don’t check at all	9	7.3	1	1.1	9	4.9	
Other	5	4.0	3	3.2	11	6.5	
Who confirms the wish of treatment to the patient? ^a^							
Intensivist	84	67.7	9	15.3	70	38.3	
Surgeon	114	91.9	55	93,2	165	90.2	
ICU nurse	29	23.4	10	16.9	79	43.2	
Palliative care team	8	6.5	3	5.1	7	3.8	
Other	2	1.6	1	2	7	3.8	
What do you discuss with the patient? ^a^							
Choice of treatment	107	86.3	52	88.1	159	86.9	0.784
Patients’ values	72	58.1	27	45.8	71	38.8	0.006
Goal of care	34	27.4	8	13.6	47	25.7	0.095
Desired place for recover	35	28.2	18	30.5	41	22.4	< 0.001
Selection of a proxy decision-maker	31	25.0	11	18.6	25	13.7	0.104
Predicted course of the disease	100	80.6	47	79.7	112	61.2	< 0.001
Prognosis	73	58.9	39	66.1	63	34.4	< 0.001
Whether the patient wants to know the prognosis and condition	42	33.9	18	30.5	66	36.1	0.132
Don’t check at all	5	4.0	1	1.7	5	2.7	0.180
Other	2	1.6	0	0.0	2	1.1	
Can the wishes of the patient regarding treatment be incorporated in the treatment strategy?							0.378
Never	0	0.0	1	1.7	5	2.7	
Occasionally	17	13.7	3	5.1	33	18.0	
Sometimes	50	40.3	19	32.2	60	32.8	
Often	48	38.7	32	54.2	77	42.1	
Always	5	4.0	3	5.1	2	1.1	
Non response	4	3.2	1	1.7	6	3.3	

*ICU* Intensive care unit

NOTES. Total numbers of intensivists, *n* = 124; surgeons, *n* = 59; ICU nurses, *n* = 189.

The table depicts one-way analysis of variance results

Fifty-four (43.9%) nurses in the surgical ward reported that they often or always considered their patients’ values and wishes regarding treatment (Table 6). In addition, 80 (65.0%) floor nurses reported that they often or always shared information on their patients’ needs across wards. However, 53 (43.1%) participating floor nurses reported that they shared their patients’ needs, as well as the process that led to the decision, and 20 (18.3%) nurses in the surgical outpatient department responded that they frequently shared patient information with the floor in which the patients were admitted. Further, 73 (67.0%), 51 (46.8%), and 50 (45.9%) participants said they shared information on the surrogate decision-maker, contents of informed consent, and values and wishes of the patients regarding treatment, respectively.

**Table 6:** Floor/outpatient nurses’ awareness of decision support

	Floor nurse		Outpatient nurse	
	n	%	n	%
Do you want to discuss with the patients their values and wishes regarding treatment?				
Never	4	3.3	26	24.1
Occasionally	26	21.1	31	28.7
Sometimes	39	31.7	18	16.7
Often	47	38.2	28	25.9
Always	7	5.7	8	7.4
Whether patients’ treatment and care needs are shared among wards				
Never	0	0.0		
Occasionally	16	13.0		
Sometimes	27	22.0		
Often	62	50.4		
Always	18	14.6		
Whether patients’ treatment and care needs, including decision-making processes, are shared across wards				
Never	6	4.9		
Occasionally	26	21.1		
Sometimes	38	30.9		
Often	48	39.0		
Always	5	4.1		
Patients’ information shared among outpatients’ and inpatients’ wards				
Process leading to the patient’s decision to receive treatment			41	38.0
Informed consent			51	47.2
Surrogate decision-makers			73	67.6
The course of treatment			24	22.2
Patient’s treatment wishes and values			50	46.3
Other			9	8.3
Whether the patient’s wishes regarding treatment are included in the information provided by other hospitals and facilities				
Never			12	10.8
Occasionally			38	34.2
Sometimes			36	32.4
Often			20	18.0
Always			0	0.0
Does the hospital share information on its patients’ wishes regarding treatment from other hospitals or facilities?				
Never			7	6.3
Occasionally			21	18.9
Sometimes			20	18.0
Often			40	36.0
Always			17	15.3

NOTES. Total numbers of floor nurses, *n* = 123; outpatient nurses, *n* = 109

## Discussion

In the current study, 40% of the target hospitals provided ACP support to ICU patients, making it a significant addition to the literature which has few studies that have clarified the status of ACP support provision to ICU patients. In a survey on physicians involved in the treatment of patients undergoing cardiovascular surgery, most of them in the ICU, 85% of the physicians considered ADs to be useful, whereas 62% reported they did not discuss the ADs with their patients out of concern that such discussions might make the patients anxious or afraid [20]. In a Japanese survey on physicians working in palliative care units, 62.6% of the respondents recommended ADs; however, only approximately 30.3% assisted with the ADs of their patients [21]. This is because palliative care physicians prefer family-centred to patient-centred decision-making in end-of-life care planning [21]. In general, research on ACP and its effects has been conducted in patients with advanced cancer and in the field of palliative care [4, 22], yet ACP is not yet accepted in these fields in Japan. Indeed, although research on ACP and ADs has been conducted for about 30 years, the term “ACP “has only recently come into common use and is not widely recognized by the Japanese public. Furthermore, in Japanese culture, patients, including their families, make decisions rather than focusing on self-determination as in Europe and America. Further, support for ACP in ICU-treated patients is still under study, and relevant research has not yet revealed any definite effects. One reason why ACP support is not actively provided to critical care patients is that healthcare providers have only a low understanding of ACP. The current study confirmed this understanding by revealing that many respondents across job categories and departments did not completely understand ACP. Previous studies indicated that although they understood the necessity of ACP support for high-risk postsurgical and ICU-treated patients, they were aware of a lack of knowledge and skills to support ACP [23, 24]. The current study provides the same result and emphasises the importance of improving healthcare providers’ recognition and understanding of ACP in the future.

In addition, the proportion of healthcare providers who confirmed DNAR with patients ranged from 10 to 20%, and those who did not reconfirm DNAR following improvement in patients’ conditions ranged from 10 to 50%. According to the literature, the extent to which patients are involved in DNAR decision-making varies widely from 25% [25] to 82% [26]. Even this involvement is often complicated by various factors, such as cultural aspects, situation, hospital policy, and individual behaviour [27]. In particular, surgical ward nurses and outpatient nurses tended not to reconfirm their intentions regarding DNAR. Very often, DNAR was checked with patients only after their condition worsened. These findings suggest that a patient’s recovery may reduce the need for ACP and weaken the perception that patients want to be treated. Further, the literature reveals preferences regarding end-of-life care change for patients who recover after ICU treatment [28], as healthcare providers find it challenging to continue the discussion on patient preferences over the course of the treatment [7].

In addition, approximately 30% of the intensivists, surgeons, and ICU nurses answered that patients would select a surrogate decision-maker, and, participants exclude outpatient nurses, the surrogate was selected by family members. The ACP discussion process includes the selection of a surrogate decision-maker but it is not sufficiently done in practice; therefore, it is often too late to ask the patient for a surrogate decision-maker, since the confirmation of the patient’s intention to undergo treatment is generally made at the time of rapid disease progression. Moreover, in some cases, a healthcare provider selects a surrogate decision-maker. In principle, the selector of surrogate decision-makers is the patient. Critically ill patients may not be able to participate in decision-making in that they often lack decision-making capacity. However, this result has possibilities indicating that they may not understand how to conduct ACP [23, 24].

It may be unclear who will support or discuss the ACP with patients [29]. Some patients also do not have a surrogate. With rapid aging in the developed world, healthcare providers have more opportunities to practice end-of-life care and thus examine and address patients’ best interests. Studies evaluating the effectiveness of established ACP interventions point out that the processes, interventions, and metrics of the caregivers about ACP are inconsistent and very complex [30]. However, the ACP may share a patient’s treatment wishes and requirements with a healthcare provider or a surrogate decision-maker and are worth being discussed and addressed in advance for critical care patients at high risk of losing their decision-making capacity.

Patients who have undergone high-risk surgery and have been admitted to ICUs show willingness to discuss ACP in advance to ensure that their treatment preferences are respected and to minimise the burden on family members toward the end of their life [28]. At the same time, an increasing number of healthcare providers are positively considering the provision of ACPs support to patients [21, 31]. Therefore, in the future, the provision of ACP support to critical care patients may be positively promoted by improving healthcare providers’ knowledge and support skills. Only approximately 20–30% of ICUs and 40% of wards and outpatients considered patients’ wishes and values regarding treatment and implemented processes that realised these wishes and decisions. Further, in acute-care hospitals, especially when patients experience rapid changes in their disease conditions, their wishes regarding treatment/care and resuscitation might differ from those recorded on admission [32, 33]. In many cases, patients and physicians do not discuss resuscitation, and the associated needs of less than half the total number of patients are absent in medical records [34]. Such incidents suggest that shared decision- making processes involving physicians, patients, and healthcare providers are inadequate, raising the concern that such decision-making increases the likelihood of patients receiving unwanted treatment, as well.

Acute-care hospitals experience difficulties in deciding when and by whom their patients’ intentions should be confirmed because the length of hospitalization is short, even for patients undergoing high-risk surgeries, and it is challenging for healthcare providers to identify the intentions of patients in severe conditions [35]. Those who receive critical care are more likely to move to the end of life than those who do not [34]. If patients enter end-of-life, they should be provided high-quality medical care that satisfies their preferences. It is necessary to ask the patients to elucidate their wishes regarding treatment and sense of value in advance at the time of hospitalization or starting treatment to initiate ACP support provision [36, 37]. Instead of focusing only on end-of-life care, ACPs should encompass patients receiving all types of treatment. Many of the outcomes of ACP studies are completion of ADs and reduction in ICU admissions [22, 38]. In Japan, because the documentation of ACP and patients’ treatment preferences is not regulated, it may not be essential for patients to document ACP. Even without documentation, surrogate decisions can be made based on presumptive intent if the surrogate decision-maker or healthcare provider understands the patient’s wishes for treatment. To respect patient autonomy, the first step is to create an opportunity for patients receiving critical care to talk to family members and healthcare providers about their values and way of life.

This study has some limitations. First, the sample size of surgeons considered in this study was small. The response rate was as low at 30% for all respondents except intensivists. Second, although it was planned to investigate the status of ACP support for each job category as well as each hospital’s response, it was challenging to secure the participation of all the healthcare providers of a single hospital, which has not been adequately factored in. In addition, because ADs, designation of a surrogate decision-maker are not determined by the law in Japan, it is not always possible to observe the intentions behind ADs and DNARs; this may also be a factor in the study’s results. These are limits to the generalizability of our findings. By examining patients’ perceptions of ACP support from various roles involved in the care of patients receiving ICU treatment, future studies may help overcome the challenges in promoting ACP support.

## Conclusions

The study found that 40% of the target acute-care hospitals undertook provision of ACP support to patients receiving critical care, suggesting that most healthcare professionals lack appropriate understanding of the importance of ACP and that, in general, ACP is initiated very late after ICU admission. Further, the perception of ACP differs among healthcare providers and ACP remains an ambiguous concept. In acute-care hospitals where patients receive different kinds of treatment or the physicians who make treatment decisions change frequently, it is necessary to confirm and connect the changing feelings and thoughts of patients’ family members and healthcare providers regarding the treatment and care provided. These findings suggest the need to develop processes that consider patients’ wishes and decisions regarding treatment, records that help transition values, early education programs targeting patients, and appropriate supporters.

## Supplementary Information

**Additional file 1.**

## Data Availability

The datasets used and/or analysed during the current study are available from the corresponding author on reasonable request.

## Abbreviations

ACP: Advance care planning
AD: Advance directive
DNAR: Do not attempt resuscitation
