# Supplementary material for: Healthcare providers’ perception of advance care planning for patients with critical illnesses in acute-care hospitals: a cross-sectional study
Source: BMC Palliat Care. 2022 Jan 7;21:7. doi: 10.1186/s12904-021-00900-5 (PMC8742355; doi:10.1186/s12904-021-00900-5)
Supplement: Supplementary file 1 — Additional file 1. [file 12904_2021_900_MOESM1_ESM.docx]

**Supplemental Appendix A1**

**Survey Sheet**

I would like to ask you about advanced directives and advanced care planning for patients in intensive care unit (ICU).

**Meaning of Terms**: For the questions in this survey, we will define the terms as follows:

**Advance directives**: Patients must present by themselves their intentions for medical treatment in advance when they lose the ability to make decisions in the future.

**Advance care planning (ACP):** In preparation for future decline in decision-making ability, healthcare professionals, patients, and their families will be asked to provide future treatment, care, and life. ACP is a discussion process, and the content of the discussion includes not only the present condition and future prospects but also the values and hopes of the person, the person’s QOL and treatment intention.

I. I would like to ask you about your attitude to treat a patient in an ICU.

1. Do you confirm the patient’s intention regarding treatment?

1) Never 2) Occasionally 3) Sometimes 4) Often 5) Always

2. When do you confirm the patient’s intention regarding treatment? (Select all that apply.)

1) On admission to the ICU

2) When condition worsens

3) At the time of disease improvement

4) Before starting a new treatment

5) Don’t check at all

6) Other (Please specify)

3. Who confirms the wish of treatment to the patient?

1) Intensivists 2) Surgeons 3) ICU Nurses 4) Palliative care team

5) Other (Please specify)

4. What do you discuss with the patient? (Select all that apply.)

1) Choice of treatment 2) Patients' values 3) Goal of care 4) Desired place for recovery

5) Selection of a proxy decision-maker 6) Predicted course of the disease

7) Prognosis 8) Whether the patient wants to know the prognosis and condition

9) Don’t check at all 10) Other (Please specify)

5. Can the wishes of the patient regarding the treatment be incorporated into the treatment strategy?

1) Never 2) Occasionally 3) Sometimes 4) Often 5) Always

II. Regarding the explanation and agreement on a do not attempt resuscitation (DNAR) with the patient, we would like to ask about the ICU in your facility.

6. Do you discuss the DNAR with the patient? Please choose one from the options listed below.

1) Never 2) Occasionally 3) Sometimes 4) Often 5) Always

7. When do you discuss the DNAR with the patient? (Select all that apply)

1) Admitted in the ICU

2) When condition worsens

3) Do not check with the patient

4) At the time of hospital admission

5) During the first outpatient visit

6) Other (Please specify)

8. Do you recheck the DNAR when the patient’s condition improves?

1) Never 2) Occasionally 3) Sometimes 4) Frequently 5) Absolutely

III. I would like to ask you about your relationship with the family/surrogate decision-maker of the patient in ICU.

9. Who decides on selecting a surrogate decision-maker for the patient? (Select all that apply.)

1) Intensivists 2) Surgeons 3) ICU nurses 4) Patient 5) Patient’s families

6) Floor nurses 7) Outpatient nurses 8) Other (Please specify)

IV. I would like to ask about the introduction of advanced care planning for patients in the ICU.

10. Have you ever implemented advanced care planning for ICU patients?

1) Yes 　　2) No

11. Have you implemented advanced care planning for ICU patients in your institution?

1) Yes, 　2) No, 　3) I don't know.

12. Who discussed to start advanced care planning to the patient? (Select all that apply.)

1) Intensivists

2) Surgeons

3) Nurses

4) Palliative care team

6) Others (Please specify)

13. What kind of information regarding the patient’s decision-making or treatment desire is transitioned to the next ward or facility to which the patient is linked? (Select all that apply.)

1) Medical information, including treatment plans

2) Treatment desired by the patient

3) Desired place for recovery

4) Patient’s values and something to live for

5) Process leading to treatment decision

6) Surrogate decision-maker

7) I will not give you any information

8) Others (Please specify)

14. Do you understand ACP?

1) Never 2) Occasionally 3) Sometimes 4) Often 5) Always

15. Which of the following statements is correct regarding the content of ACP? (Select all that apply.)

1) It is carried out for people whose prognosis is that they will die within one year.

2) The healthcare provider selects the most appropriate person as the proxy decision-maker.

3) Healthcare providers ask patients about their treatment preferences; they also ask the patients to

decide.

4) Healthcare providers must always inform patients of the expected course of illness and life

expectancy (prognosis).

5) Decisions once considered should be adhered to, and treatments’ intentions should remain

unchanged.

16. If you were to support ACP to patients and their families, how confident would you be in explaining it? Please rate on a scale of 0–100.

(“Completely confident” = 100 points and “Completely unconfident” = 0 points)

**Ⅴ. Outpatient nurses and ward nurses should answer the following questions:**

17. Do you want to discuss with the patients their values and wishes regarding treatment?

1) Never 2) Occasionally 3) Sometimes 4) Often 5) Always

18. Are the patient’s treatment and care needs shared among wards?

1) Never 2) Occasionally 3) Sometimes 4) Often 5) Always

19. Are the patient’s treatment and care needs, including decision-making processes, shared across wards? 1) Never 2) Occasionally 3) Sometimes 4) Often 5) Always

20. Is patient information shared with the outpatient and inpatient wards? (Select all that apply.)

1) Process leading to the patient’s decision to receive treatment

2) Informed consent

3) Surrogate decision-makers

4) The processes of treatment

5) Patient’s treatment wishes and values

6) Other (Please specify)

21. Are the patient’s wishes regarding treatment included in the information provided by other hospitals and facilities?

1) Never 2) Occasionally 3) Sometimes 4) Often 5) Always

22. Does the hospital share information on its patients’ wishes regarding treatment from other hospitals or facilities?

1) Never 2) Occasionally 3) Sometimes 4) Often 5) Always

**VI. I would like to ask you about yourself.**

23. How old are you?

24. How many years of work experience have you had?

25. Please tell me your gender.

26. What is your rank in your institution?

　 1) Manager 2) Chief 3) Staff

27. Please tell me your specialty clinical department.

1) Gastroenterology 2) Cardiovascular 3) Thoracic surgery 4) Neurosurgery 5) Other

28. Do you have a special qualification as a nurse? (Nurse only)

1) Certified nurse 2) Certified nurse specialist 3) Nurse practitioner 4) None

29. Please tell me the management system of the ICU.

1) Closed-ICU 2) Semi-Closed ICU 3) Open-ICU
